# Acoustic Resonance Testing of Small Data on Sintered Cogwheels

**DOI:** 10.3390/s22155814

**Published:** 2022-08-04

**Authors:** Yong Chul Ju, Ivan Kraljevski, Heiko Neunübel, Constanze Tschöpe, Matthias Wolff

**Affiliations:** 1 Cognitive Material Diagnostics Project Group of Fraunhofer Institute for Ceramic Technologies and Systems IKTS, 03046 Cottbus, Germany; 2 Condition Monitoring Hardware and Software Group of Fraunhofer Institute for Ceramic Technologies and Systems IKTS, 01109 Dresden, Germany; 3 Machine Learning and Data Analysis Group of Fraunhofer Institute for Ceramic Technologies and Systems IKTS, 01109 Dresden, Germany; 4 Chair of Communications Engineering, Brandenburg University of Technology Cottbus-Senftenberg, 03046 Cottbus, Germany

**Keywords:** acoustic resonance testing (ART), deep learning, machine learning, small data, non-destructive testing (NDT)

## Abstract

Based on the fact that cogwheels are indispensable parts in manufacturing, we present the acoustic resonance testing (ART) of small data on sintered cogwheels for quality control in the context of non-destructive testing (NDT). Considering the lack of extensive studies on cogwheel data by means of ART in combination with machine learning (ML), we utilize time-frequency domain feature analysis and apply ML algorithms to the obtained feature sets in order to detect damaged samples in two ways: one-class and binary classification. In each case, despite small data, our approach delivers robust performance: All damaged test samples reflecting real-world scenarios are recognized in two one-class classifiers (also called detectors), and one intact test sample is misclassified in binary ones. This shows the usefulness of ML and time-frequency domain feature analysis in ART on a sintered cogwheel dataset.

## 1. Introduction

Since the industrial age cogwheels (the term cogwheels may be considered as gears but *not* gearboxes or bearing systems) have been indispensable components in manufacturing, e.g., the textile and automotive industries, and they still play a significant role even in this information age, e.g., robotics and aerospace. This makes developing reliable and cost-effective non-destructive testing (NDT) methods an integral part of quality control (QC).

The field involved with cogwheels is vast, and yet most work in the literature has been performed in the context of gearboxes or bearing systems [1,2,3,4,5,6,7], i.e., many gears are inside in a system and attached to each other. For such systems, the main focus of fault detection lies on a system failure dealing with conditions monitoring in lifespan analysis, which occurs mainly due to malfunctioning components suffering from wear, abrasion and pollution, such as sand or lubrication.

However, this necessarily leads to different directions of research, when the structural health diagnosis of cogwheels in manufacturing process, e.g., sintering, comes into focus. Moreover, this often encounters small data problems simply due to lacking data of defective parts; see, e.g., [4]. Here, small data problems refer specifically to the situation when there are not enough data available for training algorithms in machine learning (ML), which poses difficulties in various fields as, e.g., can be seen in [8].

Although gearboxes or bearing systems related work have made progress [5,6,7], usually by means of modern deep learning (DL) [9], the employed methods are *not* always applicable to small data problems as discussed in [10,11], e.g., 22 layers of GoogLeNet [12] used in [6].

Moreover, not all studies reflect real-world scenarios, e.g., the tooth of a gear is intentionally cut off [5]. In addition, many studies do *not* mention the sample size of a dataset *nor* countermeasures against overfitting whether the proposed work is suitable for small data problems, e.g., [7]. Additionally, ML methods employed in some work dealing with small data are still limited to shallow learning [3], i.e., traditional ML [10]. In order to show missing points in the field and different aspects of this study, we provide an overview of signal-based methods in Table 1.

When it comes to NDT, other than signal-based approaches, there also exist image-based methods and they have made considerable progress since modern DL-based algorithms have become part of the mainstream across most research disciplines [13,14] due mainly to the work by Krizhevsky et al. [15]. However, in this study, we focus solely on signal-based methods on the grounds that image-based approaches are not as cost-effective as signal-based ones [16], and the methods become futile when defects in images are invisible as in our case. In addition, among different ML algorithms, we call them modern when the employed approaches are involved with DL-based ones; otherwise, we call them classical.

Hence, given a summary about the matter in Table 1, apart from gearboxes or bearing systems, to the best of our knowledge, there has actually been *no* extensive study on sintered cogwheel small data using acoustic resonance testing (ART) [17] with the help of traditional and modern ML methods in the context of non-destructive testing (NDT).

**Our Contributions:** In this work, we address the aforementioned issues and intend to bridge the gap: We collect a small dataset on cogwheels and perform time-frequency domain feature analysis.

Afterwards, we apply not only classical ML algorithms but also modern DL-based ones to the obtained feature sets in the way of one-class as well as binary classification. In this way, in spite of having *small data*, our approach is able to achieve robust performance: All defective test samples reflecting real-world scenarios are recognized in two one-class classifiers (also called detectors) and one intact test sample is misclassified in binary classification. This suggests that ART can be an attractive tool on cogwheel data in QC when taking advantage of the combination of ML algorithms and time-frequency domain feature analysis.

**Paper Organization:** The paper is organized as follows: After we give a brief exposition on data acquisition and feature analysis in Section 2, we provide information on training of ML algorithms in Section 3. Then, we present the result of experiments in Section 4. Finally, the paper is closed with our concluding remarks.

## 2. Materials Furthermore, Methods

### 2.1. Data Acquisition, Measurement and Sensors

#### 2.1.1. Test Objects

In the experiment, five cogwheels (chain wheels) are examined. They are made of sintered iron and inductively hardened in surface layers. The weight of the cogwheels is approximately 140 g, the outer diameter is 79 mm, and the thickness amounts to 7–9 mm.

#### 2.1.2. Examination Setup of Objects

**Figure 1:**
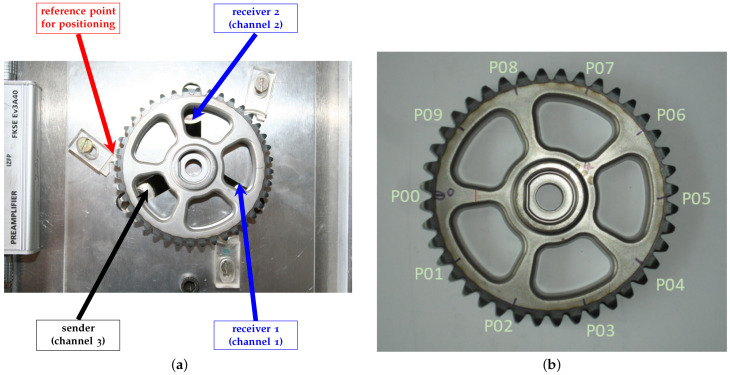
Acoustic measurement configuration of the cogwheel. (**a**) Test station for the acoustic measurement of the cogwheel; (**b**) Marked positions in the cogwheel.

The testing station for cogwheels, including a lifting device, was developed in Fraunhofer IKTS in Dresden, Germany. It is equipped with a three-point mounting system and is pneumatically controlled. In order to guarantee repeated and reproducible placement, the cogwheel is fixed when it is placed. The cogwheel is raised up to three tip points with compressed air and thus distanced from the test bench. Moreover, in these three tip points, one transmitter and two receivers (channel 1 and channel 2) are mounted, see Figure 1a.

#### 2.1.3. Measurement Method of Signal

For the measurement of signals, a multi-channel acoustic measurement system (MAS) was used: four channels, analog input amplifiers and digitization of the measurement signals, output amplifying stage for exciting acoustic converters, CAN interface to PC. In addition, two preamplifiers (40 dB; 10–500 kHz), one ultrasonic piezo actuator (transmitter) and two ultrasonic piezo sensors (receiver) are used. Each actuator and sensor is with a hard metal tip. The operating software for MAS has the following functionalities: configuring measurement channels, generating and sending excitation functions, as well as recording and storing measured signals in a time-synchronous way.

#### 2.1.4. Measurement on Cogwheels

For collecting data, the aforementioned five sintered cogwheels are used. Four of them are in intact condition, and one has defects. Concerning the defects, they were introduced by a company specialized in this area. These are designed in such a way that real-world scenarios are reflected and thereby almost indistinguishable from real ones.

For more details, we refer to [18,19,20] and the references therein. For each gear wheel, the raw signal of acoustic response that goes through a preamplifier was recorded by two receivers (channels 1 and 2) with a sampling rate of 1041.67 kHz with respect to ten different positions:

Although the receivers are mounted in fixed positions, the measurements of structural vibrations are actually obtained in different positions by rotating the wheel, which makes the data acquisition process less biased in terms of the positions of receivers. The reference point for positioning the gear is rotated in a counterclockwise direction every four teeth of the gear wheel and marked from P00 to P09, see Figure 1b. Moreover, each observation is labeled as either “OK” for intact samples or “UNK” for defective ones, respectively. The dataset is organized with respect to three excitation signals:

1. Chirp signal ranging from 1 kHz to 200 kHz (Crp1k-200k).
2. RC2 impulse with 75 kHz (RC2-75k), where RC2 is defined by RC2=0.5(1−cos(πft))cos(2πft).
3. Sinc function with 150 kHz (Sinc-150k).

**Figure 2:**
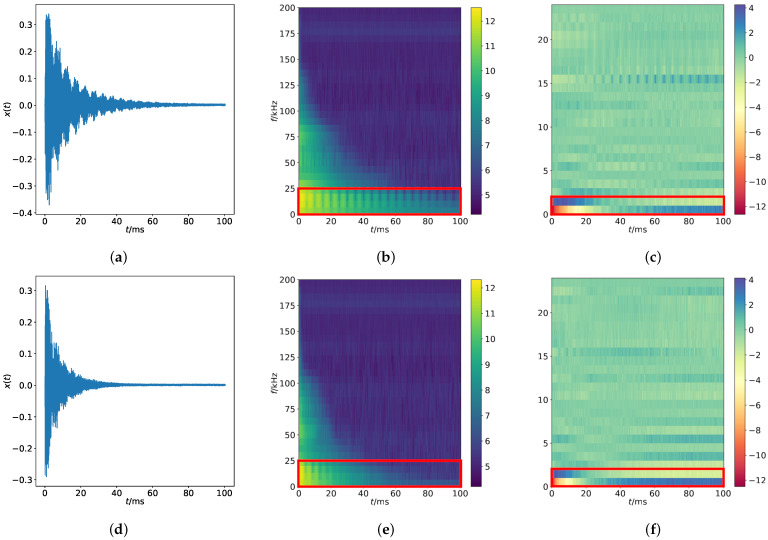
Signals of an intact and a defective sample and corresponding spectrograms of PFA and SFA for Crp1k-200k. For comparison between intact and defective samples, the most noticeable regions are marked with red boxes: (**b**) vs. (**e**) (second column) and (**c**) vs. (**f**) (third column). (**a**) Signal with an intact sample; (**b**) PFA of (**a**); (**c**) SFA of (**b**); (**d**) Signal with a defective sample; (**e**) PFA of (**d**); (**f**) SFA of (**e**).

As described in Table 2 and Table 3, there are 160 intact samples of recording and 20 defective ones for Crp1k-200k and RC2-75k and 212 intact and 20 damaged for Sinc-150k. The dimension of each observation amounts to 104,674, see Figure 2a,d.

**Figure 3:**
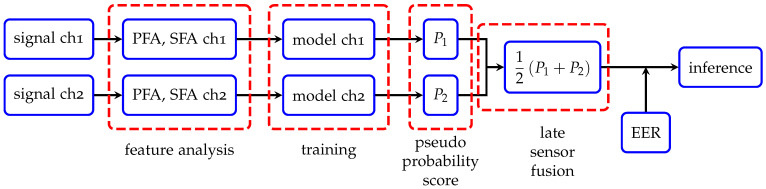
A schematic view of workflow in our approach. Channel 1 and channel 2 are abbreviated as ch1 and ch2, respectively. $P_{1}$ and $P_{2}$ denote pseudo probability scores obtained from trained models with channel 1 and channel 2, respectively.

Concerning sensor fusion methods, the late fusion approach is adopted in a sense that pseudo probability scores that are obtained from trained models using each channel are averaged to make a final prediction by incorporating the threshold of equal error rate (EER), see Figure 3. We provide more details on how the aforementioned pseudo probability scores are obtained depending on the deployed ML algorithms in Section 4.1.

### 2.2. Feature Analysis

#### 2.2.1. Primary Feature Analysis (PFA)

To perform the PFA, a short time Fourier transform (STFT) is first computed, and the resulting frequency-time dependent features are presented in the form of a spectrogram. The STFT is performed on a signal frame of Blackman analysis window with a length of 512 signal samples and MEL filter bank with a triangular function. The frame shift is 160 samples, which yields PFA features with the dimensions of 652×30; see Figure 2b,e.

#### 2.2.2. Secondary Feature Analysis (SFA)

SFA is performed based on the PFA. First, the features are rescaled to have a mean of 0 and a standard deviation of 1. Afterwards, delta features are computed by subtracting consecutive frames and principal component analysis (PCA) is performed for feature dimensionality reduction. This leads to feature vectors with a dimension of 652×24, see Figure 2c,f.

## 3. Training of Classifiers

Given the dataset, the main goal of our experiment is to investigate which combination of ML methods and feature sets are appropriate for recognizing *real-world* defects. To this end, we first considered one-class-based methods as applied in anomaly detection in order to deal with the limitations in sample size and imbalance of the acquired dataset:

- hidden Markov model (HMM),
- support-vector machine (SVM),
- isolation forest (IF), and
- autoencoder of bottleneck type (AE-BN).

Moreover, we also applied the following methods in the way of binary classification:

- feed-forward neural networks (FFNNs), and
- convolutional neural networks (CNNs).

Although NN-based methods, such as CNNs, are well-known to be useful for constructing feature maps from raw signals [9], this comes at the expensive price of a large dataset for training [21]. Moreover, this is often *not* a viable option as in our situation.

On this account, we restrict ourselves to PFA and SFA feature sets for training.

### 3.1. Configuration of Experiments

**Figure 4:**
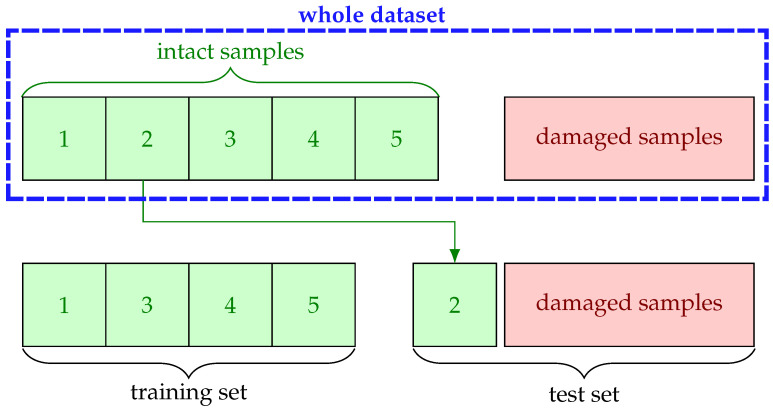
An illustration of stratified five-fold CV in terms of fold 2.

The dataset is prepared in a way that there is no overlap between training and test sets. Stratified five-fold cross validation (CV) is employed during all experiments to ensure good representation of the whole classes in the training and test folds. For one-class classification, this strategy is realized in such a way that training is performed only on intact samples without a designated fold and tested against all damaged ones with the reserved fold as illustrated in Figure 4. The reasoning behind this is to circumvent overfitting as much as possible by exploiting the common properties of small dataset, i.e., few damaged samples compared to intact ones.

#### 3.1.1. Hidden Markov Models

HMMs can be viewed as an extension of a mixture model, where the choice of the mixture component for each observation is not selected independently but depends on the choice of component for the previous observation. This is called the Markov property [22]. Since HMMs are useful for dealing with sequential data, they are widely used in speech recognition [23] and natural language processing [24].

However, they have also been successfully applied in advanced NDT [25]. Although long short-term memory (LSTM) is known to be good at dealing with variable length of sequential data [26], we instead make use of a simpler model HMM considering that our feature sets PFA and SFA have fixed dimensions. Our HMM is designed in such a way that ten hidden states release observations that correspond to our acquired dataset via one Gaussian probability density function with a full covariance matrix in each state. To detect anomalies, we used the interquartile range by measuring a score characterizing how well our model describes an observation point. The experiments are conducted by means of the dLabPro package [27], and the model parameters are estimated by the Baum–Welch algorithm [28].

#### 3.1.2. Support-Vector Machines

The SVM is a generalization of the maximal margin classifier, and it classifies data points by constructing a separating hyperplane that distinguishes one class from others [29]. SVMs are extremely powerful ML algorithms to solve various classification problems in that not only are they less prone to overfitting due to large margins but they are also relatively manageable to solve due to convex nature. Moreover, it is also well-known that they are effective in dealing with high dimensions of features—particularly when the number of features are much more than training samples—by making use of kernel tricks regarding nonlinear classification problems.

Our experiments were implemented using the scikit-learn [30] interface relying on the LIBSVM library [31]. SVM models were trained using the radial basis function (RBF) kernel, and the following parameters were tuned on about 20% of the training set to obtain optimal results: (1) regularization parameter C (from $10^{-5}$ to $10^{7}$), and (2) γ, which defines how far a single sample influences (from $10^{-10}$ to $10^{-1}$), or (3) ν, which has the ability to control over the number of support vectors (from $10^{-3}$ to 1), if necessary.

#### 3.1.3. Isolation Forest

Isolation forest belongs to the family of ensemble methods and is a tree-based anomaly detection algorithm that isolates observations as outliers based on the anomaly score delivered by profiling a randomly selected feature with a random split value between minimum and maximum values of the selected feature [32,33]. This has been a useful technique in wide range of fields, e.g., finding anomalies in hyperspectral remote sensing images [34], detecting anomalous taxi trajectories from GPS traces [35], or in analyzing partial discharge signals of a power equipment [36].

Our experiments are realized by scikit-learn [30]: the minimum split number is set to 2, and the maximum depth of each tree is defined by $\left\lceil \log_{2}n\right\rceil$, where *n* denotes the number of samples used to build the tree.

#### 3.1.4. Autoencoder of Bottleneck Type

An autoencoder refers to a type of ANN which aims at approximating original input signal in an unsupervised way [37], which is composed of two parts: encoding and decoding layers. The encoding layers are responsible for finding an efficient representation of the input vectors by learning useful features, and decoding layers attempt to reconstruct the input signal as close as possible from the acquired encoded information. Since AEs are capable of generating the compact representation of input data, which is extremely useful in terms of feature learning, there is an enormous potential to solve various problems, such as anomaly detection [38], image denoising [39] and shape recognition [40].

**Figure 5:**
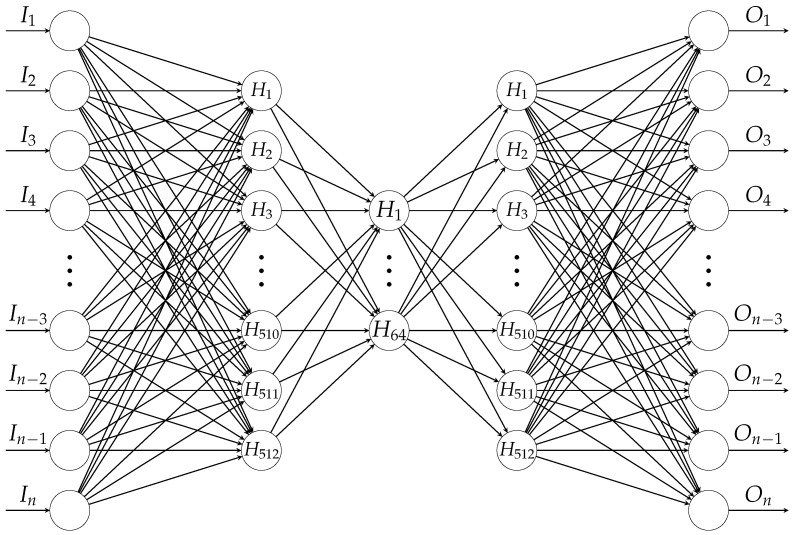
The architecture of the autoencoder of bottleneck type, where *n* denotes the dimensions: 19,560 for PFA and 15,648 for SFA.

Our experiments were performed by leveraging Keras [41] with TensorFlow [42] and the following feed-forward bottleneck type architecture is employed: input-512-64-512-output. As shown in Figure 5, the input and output size are equal to the dimensions of the vectorized feature sets, i.e., 19,560 for PFA and 15,648 for SFA, respectively.

All layers are fully connected and activated by leaky rectified linear unit (LReLU) to overcome vanishing gradient [43]. In addition, to deal with internal covariant shift *batch normalization* (BNorm) is applied to each layer [44].

Moreover, as countermeasures against overfitting, which, in our case, is of grave concern particularly due to small data, random *dropout* with a 0.5 rate in internal layers [45] and the *early stopping* strategy making use of the *patience* parameter with 25 are considered [46], where the patience specifies the number of epochs with no improvement in terms of the used loss function, after which, training will be halted [41]. Given the maximum number of epochs to be 500 in our experiments, the early stopping criterion comes into play in a range from epochs 132 to 445 depending on the folds in the datasets.

Our AE-BNs have about 20 million parameters, and for training, adaptive moment estimation (Adam) optimization [47] is incorporated along with $L_{1}$ regularization to obtain sparse solutions. Hyperparameter optimization using grid search is conducted on about 20% of training set in pre-training stages to obtain suitable parameter values, such as training batch size 512 and the aforementioned dropout rate 0.5.

#### 3.1.5. Deep Learning for Binary Classification

DL may be defined as a class of ML algorithms that typically make use of multilayer NNs in order to progressively extract different levels of representations of input data, which correspond to a hierarchy of features [48]. While the input data are being processed in multiple layers, each layer allows to reveal additional features of the input in such a way that higher level features are described in terms of lower level ones to help understand the data. As in [49], this can be understood in the following example from image classification:

Given an image of a dog as input, for instance, pixel values are detected in the first layer; edges are identified in the second layer; combinations of edges and other complex features based on the edges from the previous layer are identified in next several layers; and finally the input image is recognized as a dog in output.

**Figure 6:**
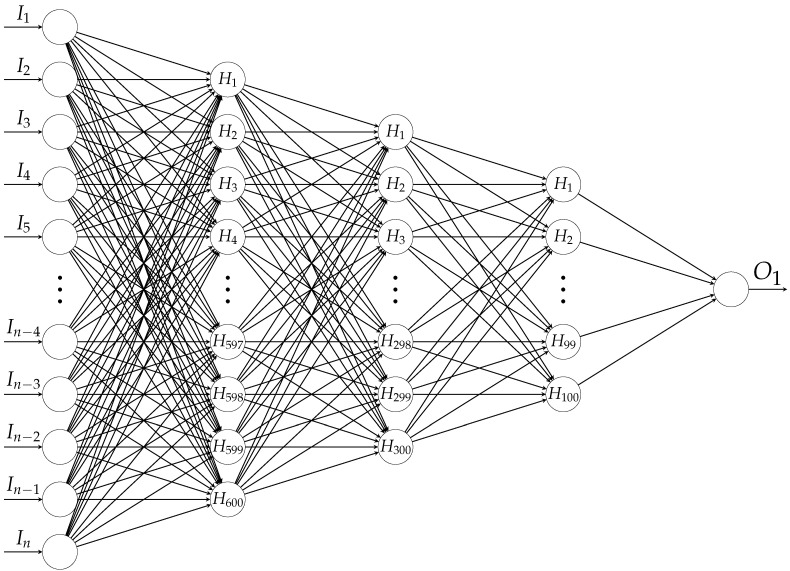
The architecture of SNN, where *n* denotes the dimensions: 19,560 for PFA and 15,648 for SFA.

Apart from the different levels of abstraction, due to the capability of nonlinear information processing, DL-based approaches have recently become popular in many fields, including, but not limited to, image processing, computer vision, speech recognition, and natural language processing [50]. As in the case of AE-BN, our DL routines were also realized by Keras [41] using TensorFlow [42], and the following architectures were employed: Three hidden layers are stacked and fully connected, see Figure 6. These hidden layers are incorporated with 600, 300 and 100 nodes and activated by the LReLU function. In addition, BNorm and a dropout rate of 0.5 are employed in each layer. Other configurations are similar to those of AE-BE: The Adam optimizer along with $L_{1}$ regularization, early stopping by means of the patience parameter with 25, batch size with 256 and maximum number of epochs with 200 are considered. From one node in the output layer, binary classification is realized using binary cross-entropy loss by mapping “UNK” to 0 and “OK” to 1. Our FFNN has about 12 million parameters.

In the case of CNN, three 2-D convolution layers with the kernel size of 3×3 are employed, each of which has 16, 32 and 64 feature maps and is downsampled with the stride of 2×2. Then, the LReLU activation function, BNorm and dropout rate with 0.75 and a 2-D max pooling layer with 2×2, which is another way to deal with overfitting, are applied to each layer. Then, the result is flattened and fed into a fully connected layer with 50 nodes activated by LReLU, where BNorm and the dropout rate 0.75 are also used.

As can be noticed, a relative high dropout rate is chosen for reducing model complexity in the light of overfitting owing to the small size of defective samples. Compared to the case of FFNN, other configurations for training remain unchanged except the maximum number of epochs at 300. The architecture of CNN is provided in Table 4. Binary classification is implemented in the same way as in the case of FFNN. Our CNN has approximately sixty thousand parameters.

## 4. Results and Discussion

### 4.1. Evaluation Metrics

In order to evaluate different classification algorithms, we provide the following performance metrics: balanced accuracy rate (BAR) along with corresponding 95% confidence interval (CI) [51], area under curve (AUC), Matthews correlation coefficient (MCC) [52] and the histogram of scores computed by one-class classifiers along with a classification margin (CM) if classes are clearly separable, i.e., if EER equals to 0.

Since scores are close to 0 and 1 for defective and intact classes, respectively, CM is defined by

(1) $$\mathrm{CM}=\frac{\min\left(S_{\mathrm{OK}}\right)-\max\left(S_{\mathrm{UNK}}\right)}{\max\left(S_{\mathrm{OK}}\right)-\min\left(S_{\mathrm{UNK}}\right)}\times 100\%\;,\;$$

where $S_{\mathrm{UNK}}$ and $S_{\mathrm{OK}}$ denote the scores of the classes “UNK” and “OK” and max(·) and min(·) stand for the maximum and minimum of the scores of the designated class, respectively.

This measure represents a ratio of a maximum margin of scores between classes to the whole spectrum of scores from both classes, where a maximum margin of scores can be computed by subtracting the maximum score of the defective class from the minimum score of the intact class.

To make an inference of a class c∈{OK,UNK} for a test set, the aforementioned scores for each detector are defined and computed based on [53] in the following way:

- HMM:
  (2) $$S_{c}:=P\left(c|x\right)=10^{-\frac{\mathrm{NLL}(x)}{\gamma }}\;\mathrm{for}\;x\in D_{\mathrm{test}}\;,$$
  where $D_{\mathrm{test}}$ denotes a test set, NLL stands for the negative log likelihood, and $\gamma =\frac{1}{\left|D_{\mathrm{test}}\right|}\sum_{x\in D_{\mathrm{test}}}^{}\mathrm{NLL}\left(x\right)$ with |·| as the cardinality of a set.
- SVM:
  (3) $$S_{c}:=P\left(c|x\right)=\frac{1}{1+e^{-\mathrm{score}(x)}}\;\mathrm{for}\;x\in D_{\mathrm{test}}\;,$$
  where score(x) denotes the distance from *x* to the separating hyperplane.
- IF:
  (4) $$S_{c}:=P\left(c|x\right)=\frac{1}{1+e^{-\mathrm{score}(x)}}\;\mathrm{for}\;x\in D_{\mathrm{test}}\;,$$
  where score(x) is defined as in [33].
- AE-BN:
  (5) $$S_{c}:=P\left(c|x\right)=\frac{1}{1+e^{-\mathrm{score}(x)}}\;\mathrm{for}\;x\in D_{\mathrm{test}}\;,$$
  where score(x) denotes the mean squared error (MSE) of the cross entropy loss function.

### 4.2. One-Class Classification

When it comes to one-class classification, despite small and imbalanced data, SVM and AE-BN perform equally well in terms of BAR across all feature sets and all three excitation functions, see Table 5. Please note that the given BAR and CI are based on beta distribution, which necessarily leads to asymmetric CIs and slightly lower values of BAR than the conventional accuracy although all test samples are correctly classified in the case of SVM and AE-BN.

In contrast to SVM and AE-BN, HMM has some difficulties with SFA in all three datasets. Moreover, when PFA is combined with either HMM or IF in the Sinc-150k dataset two misclassifications occur: intact samples are recognized as damaged ones, which is less severe than the opposite situation in a production line. The result of BAR is consistent in terms of MCC, see Table 5 and Table 6. However, AUC scores tend to be higher particularly for the case of binary classification in spite of the occurrence of one misclassification, see Table 5 and Table 7.

**Figure 7:**
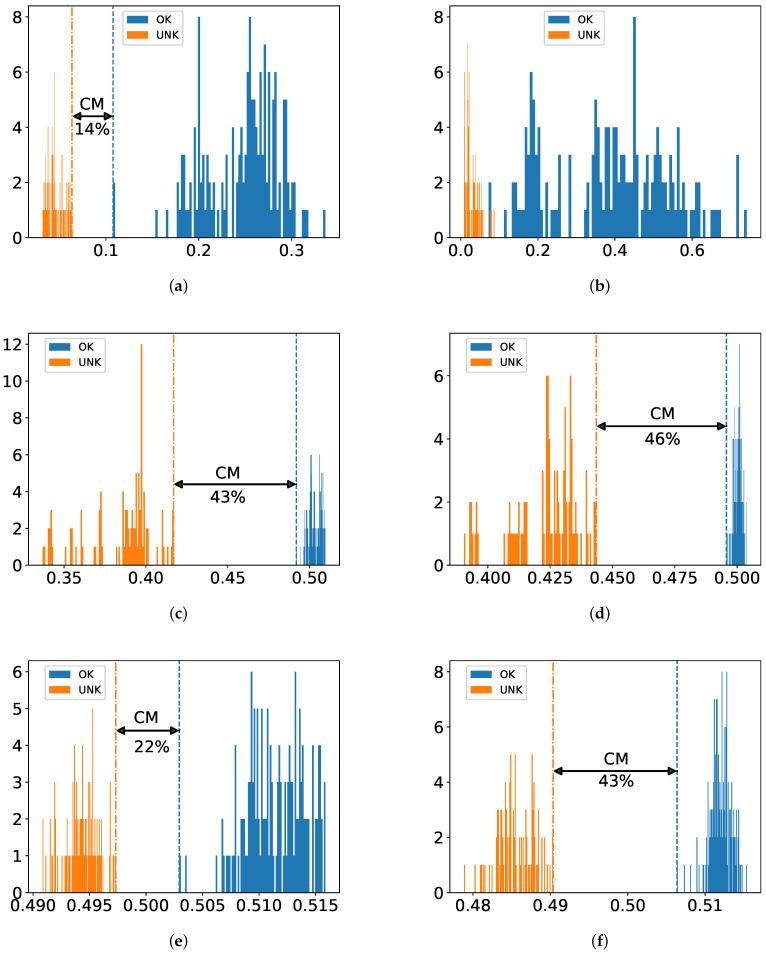

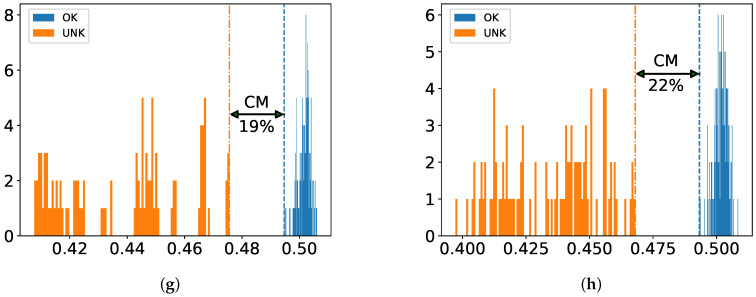
Histogram of one-class classifiers with the dataset Crp1k-200k. CM in Equation (1) is reported if available. Values on the *x*-axis are normalized classifier scores according to Equations (2)–(5). (**a**) HMM with PFA; (**b**) HMM with SFA; (**c**) SVM with PFA; (**d**) SVM with SFA; (**e**) IF with PFA; (**f**) IF with SFA. (**g**) AE-BN with PFA; (**h**) AE-BN with SFA.

**Figure 8:**
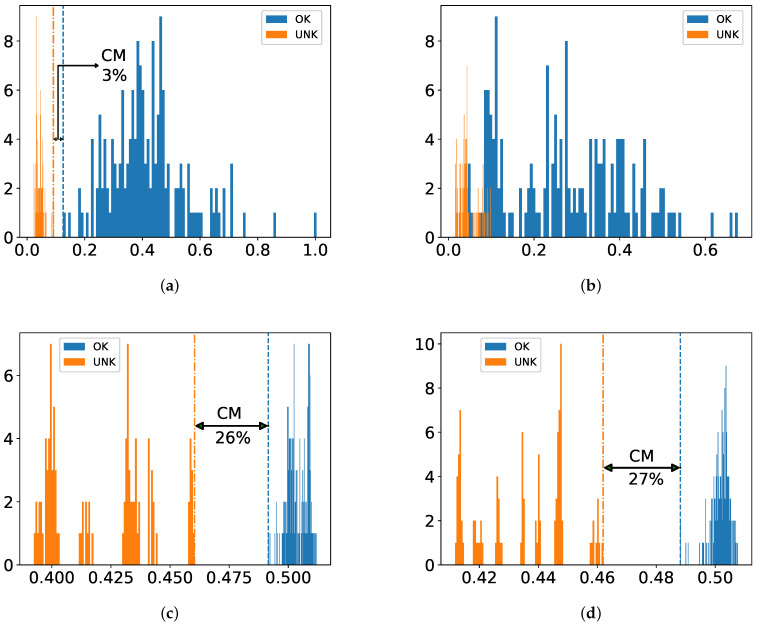

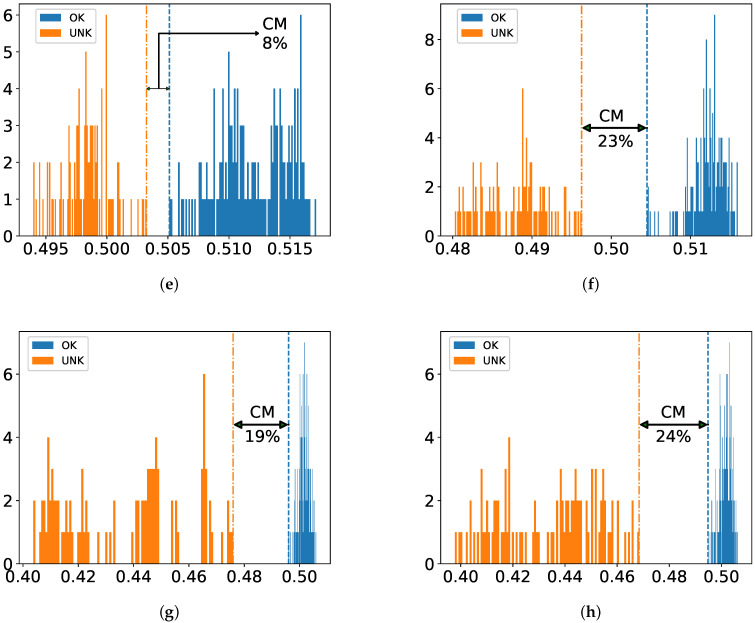
Histogram of one-class classifiers with the dataset RC2-75k. CM in Equation (1) is reported if available. Values on the *x*-axis are normalized classifier scores according to Equations (2)–(5). (**a**) HMM with PFA; (**b**) HMM with SFA; (**c**) SVM with PFA; (**d**) SVM with SFA; (**e**) IF with PFA; (**f**) IF with SFA; (**g**) AE-BN with PFA; (**h**) AE-BN with SFA.

**Figure 9:**
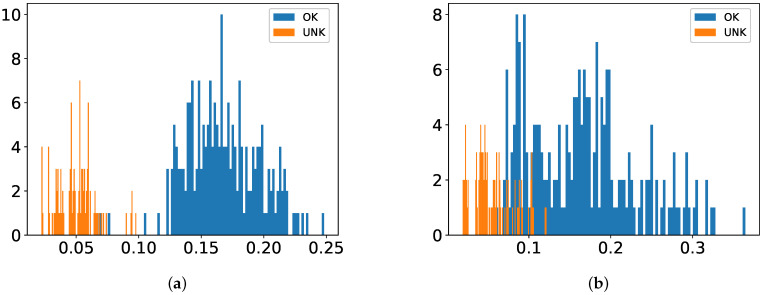

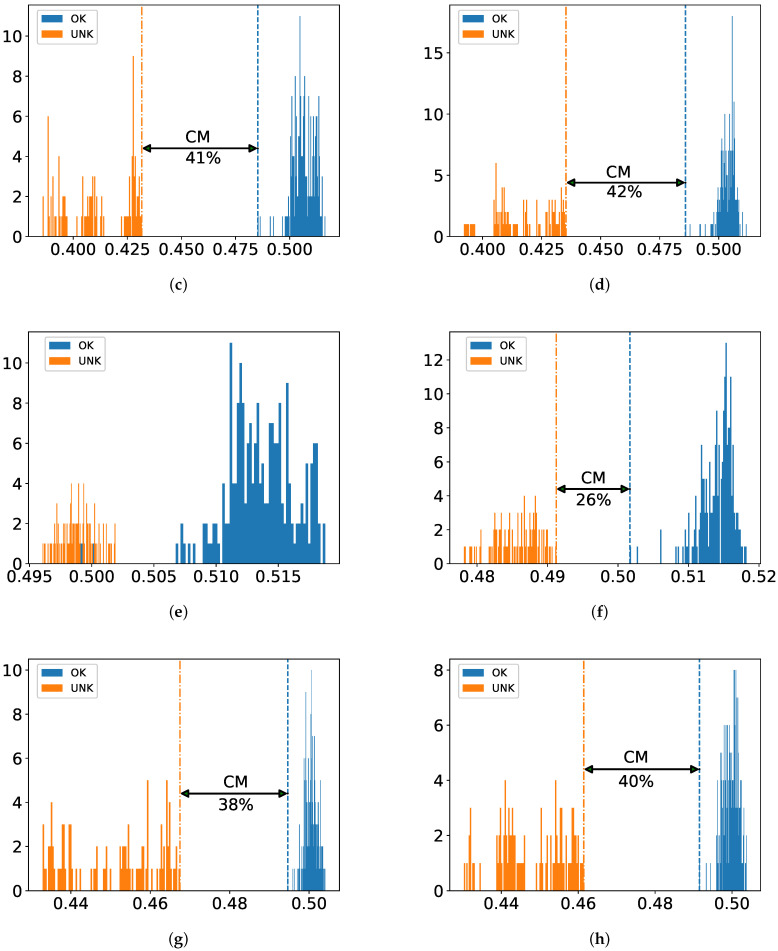
Histogram of one-class classifiers with the dataset Sinc-150k. CM in Equation (1) is reported if available. Values on the *x*-axis are normalized classifier scores according to Equations (2)–(5). (**a**) HMM with PFA; (**b**) HMM with SFA; (**c**) SVM with PFA; (**d**) SVM with SFA; (**e**) IF with PFA; (**f**) IF with SFA; (**g**) AE-BN with PFA; (**h**) AE-BN with SFA.

As shown in Figure 7, Figure 8 and Figure 9, the juxtaposed histograms of scores with the help of a CM allow us to further investigate how well classifiers behave with respect to feature sets, thresholds and robustness. The CM is available as long as classes are not overlapped.

From Figure 7d, Figure 8d and Figure 9d, one can notice that among all combinations between classifiers and feature types SVM with SFA delivers a best performance in terms of CM, which is followed by SVM with PFA, AE-BN with SFA and AE-BN with SFA in each dataset. It can be also recognized that IF gives better performance with SFA than with PFA in all databases, which, however, is not the case with HMM or AE-BN. From the perspective of excitation functions, more classifiers are able to recognize all test sets correctly in the dataset Crp1k-200k and RC2-75k than in Sinc-150k.

The result of our approach suggests that one-class classification still allows for reliable anomaly detection even though training is performed only on intact samples. Moreover, our proposed method gives robust performance by showing fairly large CM not only with classical methods but also with modern DL-based ones, e.g., 46% of SVM with SFA and 40% of AE-BN with SFA as shown in Figure 7d and Figure 9h. This makes an important point of our contribution since real-world scenarios of data skewness in a production line, i.e., numerous intact samples but few damaged ones, are considered.

### 4.3. Binary Classification

While one-class-based experiments show different results depending on combinations of classifiers and feature sets in each dataset, binary classification experiments yield one misclassification in all cases: an intact sample is misclassified as the damaged one, see Table 5. It should be noted that binary classifications, in contrast to the one-class case, make use of not only intact samples but also defective ones for training.

Since the number of flawed samples are much less than that of flawless ones, obtained models from training are prone to overfitting, which forces us to take various countermeasures, such as less complex NN architectures, high dropout rates and a higher weight of regularization. Although FFNN and CNN deliver solid performance in our case, it should be noted that it may sometimes be difficult to deal with small data.

To improve the overall performance of binary classification, it is therefore desirable to provide more data of faulty samples. In this context, data augmentation by considering the physical properties of cogwheels, e.g., numerical simulation, may be a possible approach to deal with the difficulties.

## 5. Conclusions

In this article, we presented the ART approach on small data of sintered cogwheels by utilizing not only classical ML algorithms but also modern ones. In consideration of data imbalances, our experiments were performed in two ways: one-class classification and binary classification. Our experimental results with a large safety margin classification demonstrated that one-class classifiers (detectors) had considerable potential to serve as an effective and thereby attractive tool in a reliable anomaly detection system in NDT. In addition, the experiments of binary classifiers support that they were still able to deliver robust performance in spite of small data. This shows the usefulness of ML along with time-frequency domain feature analysis on the cogwheel dataset in ART for QC.

## Figures and Tables

**Table 1:** Comparison of gearboxes, bearings, and cogwheels related work, where NM stands for “not mentioned. The symbols “🗸” and “–” denote “being affirmative” and “not applicable”, respectively.

	Qu et al. [1]	Haidong et al. [2]	Oh et al. [3]	Saufi et al. [4]	Usman et al. [5]	König et al. [6]	Žvirblis et al. [7]	This Work
Gearboxes or Bearings	🗸	🗸	🗸	🗸	🗸	🗸	🗸	–
Cogwheels	–	–	–	–	–	–	–	🗸
Shallow Learning	NM	🗸	🗸	🗸	🗸	NM	NM	🗸
Deep Learning	NM	🗸	–	🗸	–	🗸	🗸	🗸
Sample Size	NM	NM	300	750	5000	NM	NM	180–232
Small Data	–	–	🗸	🗸	–	–	–	🗸

**Table 2:** Sample size of Crp1k-200k and RC2-75k, where Z01–Z05 refers to the number of cogwheels.

	Z01	Z02	Z03	Z04	Z05	Total
intact (OK)	100	20	20	20		160
defective (UNK)					20	20

**Table 3:** Sample size of Sinc-150k, where Z01–Z05 refers to the number of cogwheels.

	Z01	Z02	Z03	Z04	Z05	Total
intact (OK)	152	20	20	20		212
defective (UNK)					20	20

**Table 4:** The architecture of CNN. The reported dimensions are for PFA.

No.	Layer Type	Filter Size	Kernel Size	Stride	Input Size	Output Size
1	Conv2D	16	3×3	2×2	652×30×1	326×15×16
2	MaxPooling		2×2	2×2	326×15×16	163×8×16
3	Conv2D	32	3×3	2×2	163×8×16	82×4×32
4	MaxPooling		2×2	2×2	82×4×32	41×2×32
5	Conv2D	64	3×3	2×2	41×2×32	21×1×64
6	MaxPooling		2×2	2×2	21×1×64	11×1×64
7	Flatten				11×1×64	704
8	Fully-connected				704	50
9	Fully-connected				50	1

**Table 5:** BAR (in percent) along with 95% CI of classifiers depending on feature sets and datasets. For each case of classifiers, the best performing ones with respect to excitation signals and used features are denoted in boldface.

	Crp1k-200k		RC2-75k		Sinc-150k	
	PFA	SFA	PFA	SFA	PFA	SFA
			Detectors			
HMM	$99.25_{-1.47}^{+0.73}$	$98.09_{-2.02}^{+1.28}$	$99.25_{-1.47}^{+0.73}$	$93.17_{-3.45}^{+2.75}$	$98.83_{-1.56}^{+0.89}$	$86.36_{-4.29}^{+3.76}$
SVM	$99.25_{-1.47}^{+0.73}$	$99.25_{-1.47}^{+0.73}$	$99.25_{-1.47}^{+0.73}$	$99.25_{-1.47}^{+0.73}$	$99.32_{-1.40}^{+0.65}$	$99.32_{-1.40}^{+0.65}$
IF	$99.25_{-1.47}^{+0.73}$	$99.25_{-1.47}^{+0.73}$	$99.25_{-1.47}^{+0.73}$	$99.25_{-1.47}^{+0.73}$	$98.83_{-1.56}^{+0.89}$	$99.32_{-1.40}^{+0.65}$
AE-BN	$99.25_{-1.47}^{+0.73}$	$99.25_{-1.47}^{+0.73}$	$99.25_{-1.47}^{+0.73}$	$99.25_{-1.47}^{+0.73}$	$99.32_{-1.40}^{+0.65}$	$99.32_{-1.40}^{+0.65}$
			Binary Classifiers			
FFNN	$97.13_{-5.86}^{+2.49}$	$97.13_{-5.86}^{+2.49}$	$97.13_{-5.86}^{+2.49}$	$97.13_{-5.86}^{+2.49}$	$97.28_{-5.81}^{+2.39}$	$97.28_{-5.81}^{+2.39}$
CNN	$97.13_{-5.86}^{+2.49}$	$97.13_{-5.86}^{+2.49}$	$97.13_{-5.86}^{+2.49}$	$97.13_{-5.86}^{+2.49}$	$97.28_{-5.81}^{+2.39}$	$97.28_{-5.81}^{+2.39}$

**Table 6:** Matthews correlation coefficient (MCC) of classifiers depending on feature sets and datasets.

	Crp1k-200k		RC2-75k		Sinc-150k	
	PFA	SFA	PFA	SFA	PFA	SFA
			Detectors			
HMM	1.0000	0.9757	1.0000	0.8714	0.9855	0.7138
SVM	1.0000	1.0000	1.0000	1.0000	1.0000	1.0000
IF	1.0000	1.0000	1.0000	1.0000	0.9855	1.0000
AE-BN	1.0000	1.0000	1.0000	1.0000	1.0000	1.0000
			Binary Classifiers			
FFNN	0.9728	0.9728	0.9728	0.9728	0.9736	0.9736
CNN	0.9728	0.9728	0.9728	0.9728	0.9736	0.9736

**Table 7:** Area under curve (AUC) of classifiers depending on feature sets and datasets.

	Crp1k-200k		RC2-75k		Sinc-150k	
	PFA	SFA	PFA	SFA	PFA	SFA
			Detectors			
HMM	1.0000	0.9994	1.0000	0.9885	0.9993	0.9625
SVM	1.0000	1.0000	1.0000	1.0000	1.0000	1.0000
IF	1.0000	1.0000	1.0000	1.0000	0.9973	1.0000
AE-BN	1.0000	1.0000	1.0000	1.0000	1.0000	1.0000
			Binary Classifiers			
FFNN	1.0000	1.0000	1.0000	1.0000	1.0000	1.0000
CNN	1.0000	1.0000	1.0000	1.0000	1.0000	1.0000

## Data Availability

Not applicable.

## References

[1] Qu Y., He D., Yoon J., Van Hecke B., Bechhoefer E., Zhu J. (2014). Gearbox Tooth Cut Fault Diagnostics Using Acoustic Emission and Vibration Sensors—A Comparative Study. Sensors.

[2] Haidong S., Hongkai J., Xingqiu L., Shuaipeng W. (2018). Intelligent fault diagnosis of rolling bearing using deep wavelet auto-encoder with extreme learning machine. Knowl.-Based Syst..

[3] Oh S.W., Lee C., You W. Gear Reducer Fault Diagnosis Using Learning Model for Spectral Density of Acoustic Signal. Proceedings of the 2019 International Conference on Information and Communication Technology Convergence (ICTC).

[4] Saufi S.R., Ahmad Z.A.B., Leong M.S., Lim M.H. (2020). Gearbox Fault Diagnosis Using a Deep Learning Model With Limited Data Sample. IEEE Trans. Ind. Inform..

[5] Usman M., Anwar S., Akmal M., Hafeez A. AI Detect: A Machine Learning Based Approach for Fault Identification in Gear Bearing System using Low-Frequency Data. Proceedings of the 2020 14th International Conference on Open Source Systems and Technologies (ICOSST).

[6] König F., Sous C., Ouald Chaib A., Jacobs G. (2021). Machine learning based anomaly detection and classification of acoustic emission events for wear monitoring in sliding bearing systems. Tribol. Int..

[7] Žvirblis T., Petkevičius L., Vaitkus P., Šabanovič E., Skrickij V., Kilikevičius A. Investigation of Deep Neural Networks for Hypoid Gear Signal Classification to Identify Anomalies. Proceedings of the 2020 IEEE eighth Workshop on Advances in Information, Electronic and Electrical Engineering (AIEEE).

[8] Zhang Y., Ling C. (2018). A strategy to apply machine learning to small datasets in materials science. npj Comput. Mater..

[9] Goodfellow I., Bengio Y., Courville A. (2016). Deep Learning.

[10] Saufi S.R., Ahmad Z.A.B., Leong M.S., Lim M.H. (2019). Challenges and Opportunities of Deep Learning Models for Machinery Fault Detection and Diagnosis: A Review. IEEE Access.

[11] Brigato L., Iocchi L. A Close Look at Deep Learning with Small Data. Proceedings of the 2020 25th International Conference on Pattern Recognition (ICPR).

[12] Szegedy C., Liu W., Jia Y., Sermanet P., Reed S., Anguelov D., Erhan D., Vanhoucke V., Rabinovich A. Going deeper with convolutions. Proceedings of the 2015 IEEE Conference on Computer Vision and Pattern Recognition (CVPR).

[13] LeCun Y., Bengio Y., Hinton G. (2015). Deep Learning. Nature.

[14] Schmidhuber J. (2015). Deep learning in neural networks: An overview. Neural Netw..

[15] Krizhevsky A., Sutskever I., Hinton G.E., Pereira F., Burges C., Bottou L., Weinberger K. (2012). ImageNet Classification with Deep Convolutional Neural Networks. Advances in Neural Information Processing Systems.

[16] Kraljevski I., Duckhorn F., Ju Y.C., Tschöpe C., Richter C., Wolff M. Acoustic Resonance Recognition of Coins. Proceedings of the 2020 IEEE International Instrumentation and Measurement Technology Conference (I2MTC).

[17] Coffey E. Acoustic resonance testing. Proceedings of the 2012 Future of Instrumentation International Workshop (FIIW).

[18] Kemppainen M., Virkkunen I. (2011). Crack characteristics and their importance to NDE. J. Nondestruct. Eval..

[19] Koskinen A., Leskelä E. Differences in different indications of three artificially produced defects in ultrasonic inspection. Proceedings of the BALTICA IX—International Conference on Life Management and Maintenance for Power Plants.

[20] Koskinen T., Virkkunen I., Siljama O., Jessen-Juhler O. (2021). The Effect of Different Flaw Data to Machine Learning Powered Ultrasonic Inspection. J. Nondestruct. Eval..

[21] Feng S., Zhou H., Dong H. (2019). Using deep neural network with small dataset to predict material defects. Mater. Des..

[22] Bishop C.M. (2006). Pattern Recognition and Machine Learning.

[23] Jelinek F. (1998). Statistical Methods for Speech Recognition.

[24] Manning C., Schütze H. (1999). Foundations of Statistical Natural Language Processing.

[25] Tschöpe C., Wolff M. (2009). Statistical Classifiers for Structural Health Monitoring. IEEE Sens. J..

[26] Hochreiter S., Schmidhuber J. (1997). Long Short-Term Memory. Neural Comput..

[27] Wolff M. (2014). dLabPro: A Signal Processing and Acoustic Pattern Recognition Toolbox. https://github.com/matthias-wolff/dLabPro.

[28] Baum L.E., Petrie T. (1966). Statistical Inference for Probabilistic Functions of Finite State Markov Chains. Ann. Math. Stat..

[29] Schölkopf B., Platt J.C., Shawe-Taylor J.C., Smola A.J., Williamson R.C. (2001). Estimating the Support of a High-Dimensional Distribution. Neural Comput..

[30] Pedregosa F., Varoquaux G., Gramfort A., Michel V., Thirion B., Grisel O., Blondel M., Prettenhofer P., Weiss R., Dubourg V. (2011). Scikit-Learn: Machine Learning in Python. J. Mach. Learn. Res..

[31] Chang C.C., Lin C.J. (2011). LIBSVM: A Library for Support Vector Machines. ACM Trans. Intell. Syst. Technol..

[32] Liu F.T., Ting K.M., Zhou Z.H. Isolation Forest. Proceedings of the 2008 Eighth IEEE International Conference on Data Mining.

[33] Liu F.T., Ting K.M., Zhou Z.H. (2012). Isolation-Based Anomaly Detection. ACM Trans. Knowl. Discov. Data.

[34] Li S., Zhang K., Duan P., Kang X. (2020). Hyperspectral Anomaly Detection With Kernel Isolation Forest. IEEE Trans. Geosci. Remote Sens..

[35] Zhang D., Li N., Zhou Z.H., Chen C., Sun L., Li S. IBAT: Detecting Anomalous Taxi Trajectories from GPS Traces. Proceedings of the 13th International Conference on Ubiquitous Computing, Association for Computing Machinery.

[36] Wang Y.B., Chang D.G., Qin S.R., Fan Y.H., Mu H.B., Zhang G.J. (2020). Separating Multi-Source Partial Discharge Signals Using Linear Prediction Analysis and Isolation Forest Algorithm. IEEE Trans. Instrum. Meas..

[37] Hinton G.E., Zemel R., Cowan J., Tesauro G., Alspector J. (1993). Autoencoders, Minimum Description Length and Helmholtz Free Energy. Advances in Neural Information Processing Systems.

[38] Sakurada M., Yairi T. Anomaly Detection Using Autoencoders with Nonlinear Dimensionality Reduction. Proceedings of the MLSDA 2014—Second Workshop on Machine Learning for Sensory Data Analysis.

[39] Gondara L. Medical Image Denoising Using Convolutional Denoising Autoencoders. Proceedings of the 2016 IEEE 16th International Conference on Data Mining Workshops (ICDMW).

[40] Hinton G.E., Krizhevsky A., Wang S.D. Transforming Auto-Encoders. Proceedings of the Artificial Neural Networks and Machine Learning—ICANN 2011.

[41] Chollet F. (2015). Keras. https://github.com/fchollet/keras.

[42] Abadi M., Barham P., Chen J., Chen Z., Davis A., Dean J., Devin M., Ghemawat S., Irving G., Isard M. TensorFlow: A System for Large-Scale Machine Learning. Proceedings of the 12th USENIX Conference on Operating Systems Design and Implementation.

[43] Maas A.L., Hannun A.Y., Ng A.Y. Rectifier nonlinearities improve neural network acoustic models. Proceedings of the ICML Workshop on Deep Learning for Audio, Speech and Language Processing.

[44] Ioffe S., Szegedy C. Batch Normalization: Accelerating Deep Network Training by Reducing Internal Covariate Shift. Proceedings of the 32nd International Conference on Machine Learning.

[45] Srivastava N., Hinton G., Krizhevsky A., Sutskever I., Salakhutdinov R. (2014). Dropout: A Simple Way to Prevent Neural Networks from Overfitting. J. Mach. Learn. Res..

[46] Prechelt L., Montavon G., Orr G.B., Müller K.R. (2012). Early Stopping—However, When?. Neural Networks: Tricks of the Trade.

[47] Kingma D.P., Ba J. Adam: A Method for Stochastic Optimization. Proceedings of the International Conference on Learning Representations.

[48] Deng L., Yu D. (2014). Deep Learning: Methods and Applications. Found. Trends Signal Process..

[49] Zeiler M.D., Fergus R. Visualizing and Understanding Convolutional Networks. Proceedings of the Computer Vision—ECCV 2014.

[50] Aggarwal C.C. (2006). Neural Networks and Deep Learning: A Textbook.

[51] Brodersen K.H., Ong C.S., Stephan K.E., Buhmann J.M. The Balanced Accuracy and Its Posterior Distribution. Proceedings of the 2010 20th International Conference on Pattern Recognition.

[52] Matthews B. (1975). Comparison of the predicted and observed secondary structure of T4 phage lysozyme. Biochim. Biophys. Acta Protein Struct..

[53] Kraljevski I., Duckhorn F., Tschöpe C., Wolff M. (2021). Machine Learning for Anomaly Assessment in Sensor Networks for NDT in Aerospace. IEEE Sens. J..

